# Triadic capabilities of sales personnel in power generation enterprises under China’s electricity market reform: Generation and evolution mechanisms

**DOI:** 10.1371/journal.pone.0355359

**Published:** 2026-08-10

**Authors:** Qingmin Kong, Peng Lin, Tingting Gu

**Affiliations:** 1 School of Business, Guangxi University, Nanning, China; 2 School of China Frontier Economic Research Institute, Guangxi University, Nanning, China; 3 School of Digital Commerce, Guangdong Finance&Trade Vocational College, Guangzhou, China; University of Zaragoza, SPAIN

## Abstract

China’s electricity market reform has prompted large numbers of frontline production workers in power generation enterprises to transition into sales roles. This transition gives rise to two interrelated questions that frame the present study: what capability structure transitioning personnel come to develop, and through what mechanisms this structure emerges and evolves. To address these questions, we adopt a dual-case research design—with CGN New Energy Guangxi as the focal case and CGN New Energy Shenzhen as the comparative case—and employ a qualitatively-driven mixed-methods approach that integrates grounded theory with a supportive questionnaire survey. Beyond the conventional “sales–service” ambidexterity framework, the study identifies digital capability as an independent core dimension and develops a “sales–service–digital” triadic capability model. Within each capability, a “foundation-layer / innovation-layer” progressive structure emerges, in which foundation-layer proficiency serves as a precondition for innovation-layer development. With respect to capability evolution, three differentiated pathways are observed—analytics-driven, relationship-driven, and market-driven—revealing patterns of multi-path convergence alongside path dependency. The findings further suggest that triadic capabilities are generated through a twofold mechanism of “endogenous development and contextual shaping”: individuals build their capabilities through proactive learning and cognitive transfer, while being concurrently shaped by market digitalization, organizational strategic positioning, and customer relationship complexity. Theoretically, this study extends sales–service ambidexterity theory in its compositional structure, advances dynamic capability theory at the individual level, and qualifies the linearity assumption underlying capability development. Practically, it provides a systematic basis for power enterprises to design differentiated developmental pathways for transitioning personnel.

## Introduction

China’s electricity sector is undergoing a fundamental transformation from a state-planned regime to a market-driven one. Under the traditional vertically integrated model, power generation enterprises were exclusively responsible for production, while sales remained monopolized by grid companies—creating a sharply bifurcated “production–distribution” structure. Since the issuance of Several Opinions on Further Deepening Power System Reform in 2015 [1], market-oriented reforms have progressively granted power generation enterprises both the right and the responsibility to compete in the market and to engage directly with end users. By the end of 2020, approximately 350,000 professionals were engaged in electricity trading nationwide, rising to 808,000 when energy management personnel on the consumer side are included [2]. This rapidly expanding workforce led to the official recognition of “Electricity Trader” as a new national occupation in July 2022. Notably, a substantial proportion of this workforce has transitioned from frontline production roles into sales positions, bringing strong technical familiarity with power generation but limited prior exposure to market-facing work.

This large-scale role transition places power generation enterprises under intense pressure to shift from a “production-oriented” to a “market- and service-oriented” mode of operation. Sales personnel are no longer expected merely to fulfill transactional functions; they must also develop in-depth understanding of customer electricity consumption behaviors (e.g., load curves, peak–valley patterns, energy efficiency preferences), design customized energy solutions, and conduct data-driven customer value assessment and market forecasting [3,4]. Yet, whether and how transitioning personnel actually develop the integrated set of capabilities required for these new roles—and through what mechanisms such capabilities emerge—remain insufficiently understood in existing literature. Research on sales personnel capabilities has primarily proceeded along two streams. The first focuses on single-dimensional capabilities, emphasizing fundamental selling skills such as communication, product knowledge, and negotiation [5,6]. The second advances dual-capability frameworks, holding that sales personnel require both sales and service capabilities to enhance customer relationship quality [7,8]. Together, these streams have laid an important foundation; however, three limitations warrant further attention. First, although data analytics has become a core competence for sales personnel in the digital era, it has not yet been systematically integrated into capability frameworks [9,10]. Second, prior research largely assumes a singular, linear developmental sequence (e.g., sales → service), thereby overlooking the multi-path, non-linear processes through which capabilities may emerge, particularly under “worker-to-sales” transitions. Third, how individual capability formation is jointly shaped by organizational institutions, technological infrastructure, and individual learning behaviors remains underexplored [11,12].

Addressing these gaps, the present study draws on CGN New Energy Guangxi as the focal case and CGN New Energy Shenzhen as the comparative case, employing grounded theory to investigate the constituent elements and generative mechanisms of sales personnel’s capabilities under China’s electricity market reform. The study makes three contributions. First, it extends the conventional “sales–service” duality by establishing digital capability as an independent core dimension, thereby developing a “sales–service–digital” triadic capability framework. Second, it reveals a dual generative logic of “endogenous development and contextual shaping,” enriching the application of dynamic capability theory at the individual level. Third, it extends context–capability matching theory to the distinctive institutional setting of China’s electricity reform, offering localized insights for organizational capability reconstruction in transitional economies. Practically, the findings provide a systematic basis for power enterprises to design developmental pathways and training systems for personnel transitioning from production to market-facing roles.

## Literature review and theoretical development

### Review of salesperson capability research

#### From unidimensional to ambidextrous capabilities.

Early research on salesperson capabilities centered on unidimensional competencies, including product knowledge and professional expertise [6,13], as well as the ability to build and maintain customer relationships [14,15]. These studies positioned selling-related skills as the core of salesperson competence and laid the foundation for subsequent capability research. As markets evolved, however—marked by increasingly diverse customer demands and a stronger orientation toward service [16,17]—reliance on selling skills alone proved inadequate. In response, scholars introduced the concept of Sales–Service Ambidexterity (SSA), emphasizing that salespeople need to develop both selling and service capabilities concurrently [7,18]. Subsequent research suggests that balancing these two capabilities is associated with stronger sales performance [19,20] and enables organizations to achieve competitive differentiation [21,22]. Nevertheless, the role of digital capabilities has received comparatively limited attention in this stream of research, often being treated as an auxiliary support rather than an independent core competence [9,10].

#### Toward multidimensional capability frameworks.

While the ambidextrous framework marks an important advance beyond unidimensional accounts, it still falls short of fully capturing the capability structure required of contemporary salespeople. Recent research has therefore moved toward multidimensional capability frameworks. A four-dimensional structure has been proposed, comprising proactive market orientation, brand management, new product development, and customer relationship management [23]. Synergistic effects among different capabilities have also been emphasized [24], along with the interactive effects of multidimensional dynamic capabilities [25].

Despite these advances, prior work has yet to provide a systematic understanding of how salespeople’s multidimensional capabilities are constituted, how they emerge and evolve, and how they perform differently across market contexts [11,12]. This gap is particularly pronounced in the case of frontline workers transitioning into sales roles, offering an important opportunity for the present study to investigate capability formation within the distinctive setting of China’s electricity market reform.

### Contingency perspective on salesperson capabilities

#### Core tenets of contingency theory.

Contingency theory holds that the effectiveness of organizational practices depends on the specific context in which they are enacted, rather than on universal best practices [26,27]. Building on this premise, scholars have introduced contingency thinking into sales capability research, arguing that sales capabilities should align with the environments organizations face [28,29]. Subsequent studies have further suggested that salesperson capabilities are moderated by market conditions and need to be adjusted in line with environmental differences [11,30]. Collectively, this stream of work provides a theoretical foundation for understanding why salesperson capabilities vary across contexts.

#### Contextual drivers of digital capability in sales.

Three contextual forces—market competition, organizational transformation, and customer demands—jointly elevate digital capability into an essential component of salesperson competence. First, intensifying market competition pushes firms to seek data-driven competitive advantages, which in turn demands that salespeople possess corresponding analytical capabilities [31,32]. Second, organizations must undergo digital transformation to adapt to market change, a process that gives rise to new digital capability requirements at the individual level [33,34] and concurrently shapes how individuals engage with digitalization through communication and persuasion channels [35].Third, evolving customer demands—particularly the growing complexity of customer relationships—call for more comprehensive capability structures, in which digital capability plays an increasingly central role [36,37].

Although prior work has highlighted the importance of digital capability from multiple perspectives, its specific content, generative pathways, and contextual antecedents remain insufficiently understood [12,38]. This study seeks to address this gap by examining how digital capability emerges and evolves in tandem with sales and service capabilities under China’s electricity market reform.

### Conceptualizing sales personnel digital capability

In decision science and information systems research, the construct of data-driven decision-making capability has evolved from the organizational level to the individual level. The concept was first proposed at the firm level [39], and subsequently extended to individual actors [40]. More recent work defines individual data-driven decision-making capability as the ability to systematically collect, analyze, and interpret data so as to identify patterns, anticipate trends, and reach optimized decisions [41,42].

Building on this foundation, we conceptualize sales personnel digital capability as an individual-level composite competence comprising data literacy, data acquisition, analytical processing, and decision transformation. Beyond the acquisition and analysis of data, what gives this capability its distinct character is the capacity to translate data-based insights into concrete selling and service decisions [43–45], supported by effective use of business intelligence tools that enable the full “data–insight–decision” chain to function in day-to-day market-facing work [46].

To avoid construct conflation, the boundaries between sales personnel digital capability and several adjacent concepts warrant further clarification. At the organizational level, digital infrastructures such as BI systems, CRM platforms, and data warehouses are technological resources owned by the firm [47], whereas sales personnel digital capability is the inherent capability that individuals exhibit when using, transcending, or operating independently of such tools; the two reside at the distinct levels of “organizational resource” and “individual capability.” At the individual behavioral level, data-driven selling characterizes the observable pattern of salespeople “drawing on data during transactions” [31,48], and technology use foregrounds proficiency in tool operation [49]; digital capability, by contrast, constitutes the underlying cognitive-skill foundation that supports such behaviors, with greater reflective and strategic-judgment qualities. At the system-operation level, CRM capability refers specifically to mastery of workflows within a particular customer relationship management system [9], whereas digital capability spans the broader “data–insight–decision” chain and is not bound to any single technological platform. Taken together, sales personnel digital capability is an individual-level cognitive-behavioral composite—neither a technological resource possessed by the organization, nor an observable behavioral pattern, nor proficiency in the operation of a single system.

### Theoretical framework

Taken together, research on salesperson capabilities has evolved from single-dimensional selling capabilities to sales–service ambidexterity [7,18], and is gradually moving toward multidimensional frameworks. However, how these capability dimensions emerge, evolve, and are shaped by contextual conditions remains insufficiently understood [8,12]. Drawing on a contingency perspective and the distinctive context of China’s electricity market reform, this study proposes a “sales–service–digital” triadic capability framework and employs grounded theory with case-based analysis to examine its generative pathways and contextual antecedents, thereby developing an integrated analytical framework of sales personnel capabilities.

## Research design

### Methodological approach

We adopt grounded theory as the principal methodology for this study, for three reasons. First, grounded theory aligns with the exploratory nature of our inquiry: it allows concepts and theory to emerge directly from raw data without requiring predetermined theoretical frameworks [50], which is well suited to the relatively underexplored question of how sales personnel capabilities are constituted in the present context. Second, grounded theory is well suited to the study of process and mechanism: by tracing how phenomena form and transform over time, it allows us to systematically uncover the developmental pathways and underlying mechanisms of capability evolution [51]. Third, grounded theory is contextually sensitive: it foregrounds the alignment between context and theory, enabling us to capture how policy, market, and organizational factors jointly shape salespeople’s capabilities under China’s electricity market reform. Within this overall approach, we follow the Gioia methodology, applying a three-tier analytical structure—first-order concepts (raw data coding) → second-order themes (conceptual aggregation) → aggregate dimensions (theoretical abstraction)—to ensure analytical rigor throughout the research process.

Because qualitative analysis is complemented by a questionnaire survey, this study is methodologically positioned as a sequential mixed method design driven by qualitative research (QUAL→quan) [52]. Within this design, grounded theory serves as the dominant component, responsible for building and constructing the constructs, while the survey functions as a complementary component, providing validation of qualitative findings within a larger sample. The survey is not intended for causal inference or independent hypothesis testing.

### Theoretical sampling and case selection

#### Research setting and theoretical sampling.

China’s electricity market reform offers a particularly suitable setting for the present study. The reform has dismantled the traditional monopoly structure, prompting power generation enterprises to transition from a production-oriented model to one centered on market and service. At the same time, concurrent digital transformation has substantially reshaped the role of sales personnel, while hundreds of thousands of frontline production workers have moved into sales positions—providing a dynamic empirical site for studying capability development. This setting combines transformational dynamics with broad representativeness, meeting the central requirement of theoretical sampling that cases be highly congruent with the theory under construction, thereby supporting the theoretical abstraction of sales personnel’s triadic capabilities.

#### Case selection: Logic and characteristics.

Following the principle of maximum variation [53,54], we adopt a dual-case comparative strategy with CGN New Energy Guangxi as the focal case and CGN New Energy Shenzhen as the comparative case. The two cases share organizational attributes (both are subsidiaries of the same central state-owned enterprise group), while differing systematically along three dimensions—market environment, level of digitalization, and stage of marketization. This combination allows us to examine the independence of digital capability and to explore the applicability of the triadic capability structure across distinct contexts.

Focal case: CGN New Energy Guangxi. This case represents typical attributes of renewable energy generation enterprises, with business operations spanning wind, solar, and biomass power. It is currently in an early stage of market-oriented transformation, with a substantial number of recently transitioned sales personnel—providing rich material for tracing capability development from its initial stage. Moreover, the relatively low level of regional digitalization and moderate market competition make this case a strong test of whether digital capability emerges as an independent dimension.

Comparative case: CGN New Energy Shenzhen. This case shares organizational culture and management systems with the focal case, controlling for organization-level heterogeneity. It operates in Shenzhen’s highly digitalized and intensely competitive urban market, with a more mature stage of transformation and a more developed capability system among sales personnel. Its business portfolio also includes energy storage and other highly digitalized segments, allowing further examination of digital capability under high-digitalization conditions.

The key characteristics of the two cases are summarized in Table 1.Together, they offer a meaningful contrast that supports the development of the triadic capability framework.

**Table 1:** Comparative characteristics of the dual cases.

Dimension	Focal Case (Guangxi)	Comparative Case (Shenzhen)
Business Portfolio	Diversified (wind, solar PV, biomass)	Specialized (solar PV, energy storage)
Market Environment	Low digitalization, moderate competition	High digitalization, intense competition
Customer Composition	Diverse: industrial, commercial, agricultural, residential, and government	Primarily large industrial and commercial clients
Marketization Stage	Early stage of transformation	Mature stage of transformation

### Data collection

#### Data collection strategy.

To enhance the credibility and validity of the analysis, this study employed multi-source data triangulation, drawing on three complementary sources: in-depth interviews, archival documents, and a questionnaire survey.

In-Depth Interviews (Primary Source). Semi-structured interviews were conducted with 28 participants—16 from CGN New Energy Guangxi and 12 from CGN New Energy Shenzhen—covering frontline sales personnel (50.0%), middle-level managers (35.7%), and senior managers (14.3%), so as to ensure perspective diversity. The interview protocol was organized around four thematic modules: role change perception, capability requirement adaptation, practical challenges, and development strategies, while avoiding leading questions throughout. Each interview lasted 40–60 minutes and was, with the participants’ consent, audio-recorded and transcribed verbatim to preserve the integrity of the original accounts. Participant characteristics are summarized in Table 2.

**Table 2:** Interview participant sample characteristics.

Classification Dimension	Category	Number	Percentage
Organizational Affiliation	CGN New Energy Guangxi	16	57.1%
	CGN New Energy Shenzhen	12	42.9%
Position Level	Frontline sales personnel	14	50.0%
	Middle-level managers	10	35.7%
	Senior managers	4	14.3%
Work Experience	Less than 3 years	5	17.9%
	3–5 years	8	28.6%
	6–10 years	9	32.1%
	More than 10 years	6	21.4%
Educational Background	Associate degree	4	14.3%
	Bachelor’s degree	18	64.3%
	Graduate degree or above	6	21.4%

*Note: N* = 28.

Archival Documents (Supplementary Source). Two categories of archival materials were collected to contextualize and corroborate the interview findings. Internal materials from the two case companies included training documents, performance evaluation standards, meeting minutes, and strategic planning documents, used primarily to verify the credibility of interview accounts. External materials included policy documents on China’s electricity market reform, industry reports, and market trend analyses, used to reconstruct the macro institutional and market context of the study.

Questionnaire Survey (Validation Source). Building on the preliminary coding of the interview data, a structured questionnaire was developed around three dimensions: triadic capability cognition, capability development pathways, and contextual factor perception. Each construct was measured with multi-item scales, which demonstrated acceptable internal consistency. The questionnaire was distributed to sales personnel across both companies, yielding 146 valid responses for analysis (response rate: 90.1%). The sample spans different positions and age groups, with details summarized in Table 3.

**Table 3:** Questionnaire survey sample characteristics.

Characteristic	Category	Number	Percentage
Gender	Male	96	65.8%
	Female	50	34.2%
Age	20–30 years	47	32.2%
	31–40 years	68	46.6%
	41–50 years	24	16.4%
	Above 50 years	7	4.8%
Position Level	Frontline sales personnel	94	64.4%
	Sales supervisors	31	21.2%
	Middle-level managers	16	11.0%
	Senior managers	5	3.4%
Organizational Affiliation	CGN New Energy Guangxi	78	53.4%
	CGN New Energy Shenzhen	68	46.6%

*Note: N* = 146.

### Coding procedures

Data Preparation. Interview recordings were transcribed verbatim, yielding approximately 270,000 words of textual data, while archival materials were digitized into approximately 280 pages of analyzable text. Survey data were cleaned and analyzed using SPSS (Version 26.0). All datasets—interview transcripts, archival documents, and survey records—were then imported into NVivo 14 to establish an integrated database that enabled cross-source retrieval and comparative analysis.

Coding Implementation. Following the Gioia methodology [50], we adopted a three-stage coding process consisting of open coding, axial coding, and selective coding. In the open coding stage, two researchers independently conducted line-by-line analysis of the raw data, extracting first-order concepts grounded in participants’ own expressions (e.g., “analyzing customer electricity load data,” “customizing green electricity packages”), while avoiding predetermined theoretical frameworks. During axial coding, related first-order concepts were grouped into second-order themes—for instance, concepts such as “load data analysis” and “electricity cost calculation” were consolidated under the theme of “data processing capability.” In the selective coding stage, second-order themes were further abstracted into aggregate dimensions, ultimately yielding the “sales–service–digital” triadic capability structure and its developmental mechanisms.

Coding Reliability. Several measures were implemented to ensure the reliability of the coding process. The two researchers performed cross-validation after every three transcripts, discussing discrepancies and reaching consensus throughout the analysis. Inter-coder agreement was 79.6% in the initial round and rose to 91.3% after three rounds of revision, exceeding the conventional threshold commonly applied in qualitative research. For coding disagreements that could not be resolved through bilateral discussion, a third independent researcher experienced in grounded theory was invited to serve as an arbiter, determining the final coding outcomes.

### Ethics statement

Ethical Approval. This study was conducted in accordance with the Declaration of Helsinki and received formal ethical approval from the Academic Committee of the School of Business, Guangxi University (Approval Number: 2025011301; Approval Date: January 13, 2025). The approved research protocol covered all aspects of data collection, including interviews, archival document access, and questionnaire administration. Informed Consent. Differentiated consent procedures were employed for the two principal modes of data collection, each pre-approved by the Academic Committee. For the in-depth interviews, oral informed consent was obtained from each participant prior to the interview. Oral consent—rather than written consent—was adopted because participants, as employees of the case companies, expressed concerns that signed documents might compromise the anonymity of their participation; the Academic Committee approved this consent format on the condition that the consent process be audio-recorded. Accordingly, each participant received a detailed explanation of the study’s purpose, procedures, potential risks and benefits, confidentiality measures, and the right to withdraw at any time without penalty, and provided oral consent that was documented in the opening segment of the audio recording. For the online questionnaire, a clear information statement was presented before the survey, explaining the study’s purpose, voluntary nature, and right to withdraw; completion of the questionnaire constituted implicit consent.

Participants. All participants were adult employees of the case companies (aged 20 years and above; see Table 3). The study involved no minors, patients, or other vulnerable populations.

Privacy Protection and Data Management. All research procedures strictly adhered to confidentiality protocols. Personal identifying information of participants—including names and specific job titles—was anonymized immediately upon data collection and represented in the research dataset by codes (e.g., “Interviewee #17”), thereby ensuring individual-level anonymity. Research data are stored securely in accordance with institutional data-protection policies; any residual files containing personal identifying information will be permanently destroyed within six months of the conclusion of the research.

## Analysis and findings

### Open coding

The open-coding phase aimed to extract initial concepts that captured the richness of sales personnel’s capability manifestations. As a starting point, we identified 33 critical-incident narratives from the interview data as priority coding materials, each reflecting a pivotal moment in the evolution of capability structures under market-oriented transformation. One such narrative reads:

“In mid-2022, we introduced a load analysis and forecasting system, which marked a watershed moment in our team’s data capability development. Before that, we relied on experiential judgment; after that, we began making data-driven decisions.”(Interviewee #8)

Throughout the coding process, we paid particular attention to participants’ own expressions, behavioral descriptions, and capability perceptions, in order to preserve the in vivo concepts embedded in the data and to allow theoretical categories to emerge inductively rather than from any predetermined framework. Representative examples of how raw data were transformed into initial codes are provided in Table 4

**Table 4:** Examples of raw data and initial coding.

Raw Data	Initial Coding	Coding Rationale
“Previously, I only looked at monthly electricity consumption, but now I analyze daily load curves, peak–valley distribution, and usage patterns, which has completely transformed how I communicate with clients.”	Deepening of data analytical capability	Reflects the transition from simple statistical analysis to complex pattern recognition in data analytics
“I’ve noticed that colleagues who can leverage data to predict customer needs are often better prepared in advance, and their service delivery is more comprehensive.”	Advantages derived from data prediction	Reveals the enabling effect of digital capability on service capability
“In a digitalized market like Shenzhen, customers expect to see data. But in most markets in Guangxi, relationship-based approaches may still dominate.”	Perception of market-environment differentiation	Demonstrates the moderating effect of contextual factors on capability application

Through multiple rounds of iterative coding, 197 initial codes were generated. After merging semantically similar items and eliminating redundancies, the codes were consolidated into 127 first-order concepts, which laid the foundation for the subsequent axial coding stage. The evolutionary trajectory of selected concepts is illustrated in S1 Fig.

A noteworthy observation from this phase was that a cluster of capability manifestations—related to data collection, analytical processing, and data-based decision-making—could not be adequately accommodated within the traditional “sales–service” dual framework. Several interviewees explicitly distinguished between “experience-driven” and “data-driven” modes of selling, hinting at the presence of a distinct capability dimension. This observation prompted us to revisit the theoretical boundaries of salesperson capability structures, providing a preliminary basis for the conceptual development pursued in the following stages of analysis.

### Axial coding and the triadic capability structure

#### From themes to a triadic capability structure.

Building on the 127 first-order concepts identified through open coding, we applied the constant comparative method to examine inter-conceptual relationships and to group related concepts into coherent categories (see S2 Fig). Through iterative comparison and abstraction, the 127 first-order concepts were ultimately consolidated into 24 second-order themes and then aggregated into 7 aggregate dimensions; the complete mapping from first-order concepts through second-order themes to aggregate dimensions is provided in S1 Table.

A central pattern that emerged through this process was the differentiation of three core capability dimensions: sales capability (A3), service capability (A2), and digital capability (A1). Notably, digital capability (themes N1–N4) exhibited conceptual independence: rather than serving as an auxiliary instrument for sales or service activities, its essence lies in the systematic collection, analysis, and application of data to support decisions. This independence was repeatedly articulated by interviewees themselves, as illustrated by the following account:

“Data analysis and sales experience represent entirely different mindsets. Experience tells me what a customer might need, but data analysis reveals why they need it, exactly how much they need it, and when they need it—this is what today’s market demands.”(Interviewee #17)

On this basis, we propose a “sales–service–digital” triadic capability structure, as summarized in Table 5. Boundary Distinctions among the Triadic Capabilities. Although the three capabilities reinforce one another in sales practice, their construct boundaries are clearly distinguishable along three dimensions. First, in terms of goal orientation, sales capability is directed toward “transaction completion,” service capability toward “problem-solving and relationship maintenance,” and digital capability toward “the transformation of data into decisions.” Second, in terms of action pathways, the three capabilities follow distinct value chains: “value identification–delivery–closure,” “need diagnosis–solution design–experience optimization,” and “data acquisition–analysis–decision-making,” respectively. Third, in terms of cognitive modes, interviewees explicitly distinguished among “experiential judgment,” “service-oriented thinking,” and “data-modeling thinking” as three independent ways of thinking, as exemplified by the account quoted above. Taken together, the three capabilities exist as independent dimensions while operating in synergy in everyday sales practice.

**Table 5:** Triadic capability structure of sales personnel.

Capability Dimension	Themes	Primary Manifestations	Core Attributes
**Sales capability**	N9–N12	Identification of customer value needs; articulation of distinct value propositions; transaction completion; resource integration for sales realization	Needs identification accuracy; value communication clarity; transaction efficiency; resource mobilization capacity
**Service capability**	N5–N8	Customer-centric orientation; problem diagnosis and resolution; long-term relationship maintenance; personalized solution design	Response velocity; problem-solving depth; relationship-building proficiency; solution customization
**Digital capability**	N1–N4	Systematic collection of multi-source data; analytical pattern identification; visualization-based insight presentation; transformation of data into decision-oriented actions	Data acquisition scope; analytical complexity; predictive temporal horizon; decision-making autonomy

#### The dual-layer structure within each capability.

Beyond the differentiation among the three capability dimensions, our analysis identified a further internal pattern: each of the three capabilities is internally structured as a dual-layer system, comprising a foundation layer and an innovation layer (see Table 6). This structure was repeatedly observed across the interviews. For example:

**Table 6:** The dual-layer internal structure of triadic capabilities.

Capability Dimension	Foundation Layer	Innovation Layer
**Sales capability**	Product presentation; price negotiation; standardized transaction completion	Precision customer targeting; value-based marketing; solution-oriented selling
**Service capability**	Standardized response protocols; reactive problem solving; basic need fulfillment	Personalized solution design; proactive need anticipation; collaborative value co-creation
**Digital capability**	Basic data collection; descriptive analytics; passive data utilization	Systematic data integration; predictive modeling; proactive data-driven decision-making

“Previously, I primarily compiled monthly data reports (foundation layer); now I develop predictive models to anticipate customer demand fluctuations (innovation layer).”(Interviewee #19)

“The real challenge lies in moving from ‘knowing how to do’ to ‘knowing how to think’—from process-driven customer service to proactive need anticipation and solution design.”(Interviewee #12)

The foundation layer represents the operational baseline of each capability—standardized routines through which sales personnel respond to recurring tasks. The innovation layer, by contrast, reflects higher-order extensions—predictive, proactive, and integrative practices that go beyond established routines. Crucially, the two layers are not parallel choices but progressively related: foundation-layer proficiency serves as a prerequisite for the development of innovation-layer practices. In this sense, capability development is not merely a quantitative accumulation of routine actions, but involves a qualitative shift in cognitive and behavioral patterns—from “doing the job” to “rethinking the job.”

#### Development pathways of triadic capabilities.

To unpack the dynamic processes of capability generation, we analyzed each capability development trajectory in terms of its contextual conditions, key actions, and observed outcomes. Three differentiated pathways emerged from the data, each reflecting a non-linear sequence of capability development:

(1) Analytics-Driven Pathway: digital capability → sales capability → service capability. Within the technical sales team of CGN New Energy Shenzhen, the following pattern was observed. Contextual conditions: a highly digitalized market environment, a technology-oriented organizational culture, and high accessibility of data systems. Key actions: initial establishment of an analytical foundation; use of data-based insights to inform selling activities; followed by enhanced service personalization. Observed outcomes: improved selling precision, stronger customer trust, and the formation of differentiated competitive advantages. “Initially, we could only claim that our solution was superior, but customers remained unconvinced. Then we adopted a load-analysis system that produced hourly consumption curves, which allowed us to show specific cost savings through production-schedule adjustments. Data gave us confidence; through analytics-based selling we gradually earned customer trust, and only later did we begin offering comprehensive solutions and participating in project tenders.”(Interviewee #25)
(2) Relationship-Driven Pathway: service capability → digital capability → sales capability. Within the key-account team of CGN New Energy Guangxi, an alternative pathway was identified. Contextual conditions: a complex customer-relationship environment, a service-differentiation strategy, and customer sensitivity to service quality. Key actions: building trust through high-quality service; accompanied by access to in-depth customer data that supported enhanced analytical work; and subsequently value-based selling. Observed outcomes: high-retention customer relationships, access to high-quality data, and high-value transactions. “When a client’s factory received an urgent production order requiring electricity capacity beyond their original budget, they reached out to us. We worked through the night to design a temporary supply solution priced below market levels. The trust generated through this response opened the door to a longer-term partnership: the client said that only we truly understood the challenges of manufacturers, and granted us access to their complete consumption data, which allowed us to propose more precise long-term collaboration plans.”(Interviewee #13)
(3) Market-Driven Pathway: sales capability → service capability → digital capability. Within the regional development team of CGN New Energy Guangxi, a third pathway was identified. Contextual conditions: a low-digitalization competitive market, a market-share strategic priority, and an extensive customer base with limited relationship depth. Key actions: prioritizing sales-capability development to expand the customer base; improving customer retention through enhanced service; subsequently leveraging accumulated data for decision optimization. Observed outcomes: a broad customer foundation, improved customer retention, and more efficient resource allocation. “We maintain service points across many less developed counties and townships, serving customers regardless of their consumption volume. Many field staff have little time for data analysis. After establishing a basic customer foundation, we began identifying premium customers for focused service, and later used their consumption data to identify similar enterprises for targeted outreach. This is a stepwise process; precision marketing cannot be implemented from day one.”(Interviewee #15)

These three pathways suggest that capability development among transitioning sales personnel exhibits contextual dependency and pathway diversity, extending beyond traditional linear assumptions of capability development.

### Selective coding and theoretical saturation

During the selective coding phase, we established “triadic capability” as the core category and consolidated the previous findings into an integrative framework of sales personnel triadic capability (see S3 Fig). The framework comprises three interrelated components: (i) a triadic capability structure that integrates the sales, service, and digital dimensions; (ii) a dual-layer internal structure, in which each capability comprises a foundation layer and an innovation layer; and (iii) three developmental pathways—analytics-driven, relationship-driven, and market-driven—through which the triadic capability evolves over time.

Triadic capability structure and evolutionary model for sales personnel. The three elongated ellipses represent the sales, service, and digital capabilities. The central circle represents the convergence of these three capabilities into the integrated competence of sales personnel. Dashed lines indicate the dual-layer internal structure comprising the foundation layer and the innovation layer. Arrows denote the three developmental pathways of capability evolution.

To assess theoretical saturation, we applied three complementary procedures. First, the coding of the final three interview transcripts yielded no new first-order concepts or second-order themes, indicating conceptual saturation within the data. Second, a retrospective re-examination of the full dataset confirmed that the framework was able to account for the patterns observed across all cases. Third, two external experts not involved in the original coding independently reviewed the framework against representative excerpts of the data and judged it to be theoretically saturated.

## Case re-examination and theoretical refinement

In grounded theory research, selective coding is not the endpoint of theory building but the starting point for further refinement. To examine the robustness of our findings and to position the framework in relation to existing literature, we conducted a systematic case re-examination of the triadic capability framework developed in the preceding chapter. This process pursues three goals: clarifying the theoretical positioning and conceptual boundaries of the core constructs, examining potential omissions or alternative interpretations, and deepening the understanding of how the triadic capabilities are generated.

### Case re-examination and theoretical positioning

The aim of this section is to bring the proposed framework into dialogue with established literature, in order to clarify the theoretical boundaries, contributions, and distinctions of the “triadic capability framework” relative to prior research.

#### Independence of the triadic capability structure.

The “sales–service–digital” triadic capability structure proposed here moves beyond the conventional “sales–service” dual capability framework [7,18]. Its core contribution lies in conceptualizing digital capability as an independent core dimension rather than as an auxiliary instrument of sales or service activities [9]. The theoretical distinctions among the three dimensions are summarized in Table 7 through a construct-attribute comparison. Additional evidence for the independence of digital capability comes from the coding data: themes related to digital capability (N1–N4) were explicitly mentioned by 87.5% (24/28) of interviewees, and were articulated independently of sales- and service-related themes. As Interviewee #17 explained:

**Table 7:** Theoretical attribute comparison of the triadic capability dimensions.

Capability Dimension	Core Construct Definition	Value Logic Chain	Differentiation from Existing Research
**Sales capability**	Capability for customer value identification, value proposition delivery, and transaction completion	Value insight → proposition communication → transaction realization	Differs from the traditional product-selling orientation by highlighting advanced features of “integrated energy solution selling” in a servitization context
**Service capability**	Capability for customer need diagnosis, problem resolution, and experience optimization	Need identification → solution design → satisfaction enhancement	Extends the generic service definition by emphasizing contextualized capabilities in the energy sector, such as “outage response” and “green-energy solution customization”
**Digital capability**	Capability for systematic data acquisition, analysis, and decision transformation	Data → insight → decision → value output	Differs from the organizational-level definition by focusing on operational capabilities in sales contexts, such as “customer-data interpretation” and “electricity-demand forecasting”

“Data analysis and sales skills are two different things; the former requires modeling thinking, while the latter requires communication skills.”

This pattern suggests that interviewees themselves perceive digital capability as a cognitively distinct domain, lending empirical support to its theoretical positioning as an independent dimension.

#### Alignment and extension of the dual-layer structure.

Re-examination of the cases shows that each of the three capabilities exhibits a “foundation-layer / innovation-layer” structure (see Table 8). This finding both aligns with and extends existing capability-hierarchy perspectives. It is broadly consistent with the notion of vertical capability stratification [33]: the foundation layer corresponds to operational capabilities (e.g., data collection, standardized service), whereas the innovation layer corresponds to dynamic capabilities (e.g., predictive modeling, value co-creation).

**Table 8:** Theoretical alignment and extension of the dual-layer structure.

Capability Dimension	Foundation Layer	Innovation Layer	Theoretical Alignment and Extension
**Sales capability**	Product presentation; basic negotiation	Solution-based selling; cross-functional resource integration	Extends Rapp et al.’s (2020) ambidextrous capability framework by highlighting advanced forms of sales capability in servitization contexts
**Service capability**	Standardized response; basic problem solving	Personalized solution design; value co-creation	Complements prior service-capability research by clarifying contextualized directions for innovation in energy services
**Digital capability**	Basic data collection; descriptive analytics	Predictive modeling; data-driven decision-making	Aligns with Teece’s (2020) dynamic capability theory while extending its account of how data-related capabilities transition from the operational to the innovative level

The extension introduced by this study lies in the inter-layer relationship: rather than coexisting as parallel structures, the two layers are progressively related, with foundation-layer proficiency serving as a prerequisite for innovation-layer development. As Interviewee #19 noted:

“Only after mastering basic data organization can one learn electricity load forecasting modeling.”

This observation addresses a gap in the “operational routines / dynamic capabilities” framework [55], namely the relatively underspecified mechanism through which the two layers connect.The dual-layer structure identified here suggests that capability evolution involves a transition from quantitative accumulation at the foundation layer to qualitative transformation at the innovation layer.

#### Differentiation among development pathways.

Prior research has tended to assume a singular, linear sequence of sales-personnel capability development, typically “sales → service” [8,20]. The present study identifies three differentiated pathways: analytics-driven (digital → sales → service), relationship-driven (service → digital → sales), and market-driven (sales → service → digital), each associated with distinct contextual conditions. This finding extends prior accounts in three respects.

Pathway diversity. Pathway selection appears to be contextually conditioned rather than universally optimal: highly digitalized markets are more often associated with analytics-driven pathways, while less digitalized markets tend to be associated with market-driven pathways. This pattern is broadly consistent with arguments regarding the environmental adaptability of capability development [33].

Path dependency. Initial capability choices appear to shape the efficiency of subsequent development. Analytics-driven pathways, for instance, show comparatively high efficiency in precise customer targeting but tend to require additional investment in relationship building. This pattern is broadly consistent with the notion of historical dependence in capability development [56], and points to an underlying cognitive-transfer mechanism: initial capabilities establish cognitive frames such as analytical thinking or service-oriented thinking that influence the learning efficiency of subsequent capabilities.

Multi-path convergence. Different pathways may nonetheless yield similar triadic capability structures. For example, the relationship-driven pathway at CGN New Energy Guangxi and the analytics-driven pathway at CGN New Energy Shenzhen both ultimately produced sales–service–digital capability synergies. This observation extends prior accounts of sequence relatedness in capability development [57] and motivates the proposition of “multi-path convergence.” Beyond the three pathways, the data also indicate the operation of positive feedback loops among the three capabilities. As one interviewee put it:

“Data analysis transformed our sales approach from product selling to solution selling, which deepened our service, and in turn generated more customer data.”(Interviewee #8)

Such accounts suggest that the three capabilities are not merely additive: they appear to co-evolve through functional complementarity and cognitive transfer.

In summary, the case re-examination indicates that the framework of “triadic capability structure, dual-layer internal characteristics, and multi-pathway evolution” addresses three core questions: what the capabilities are (structure), what internal features they exhibit (dual-layer organization), and how they develop over time (multi-pathway evolution). The remaining question, namely how the capabilities are generated under varying contextual conditions, is taken up in the following section through a focused re-examination of contextual factors.

### Generative factors of the triadic capabilities

This section turns to the question of how the triadic capabilities are generated under varying contextual conditions. Drawing on the interview, survey, and archival data, we identify two interrelated generative pathways: endogenous development driven by individual agency, and external shaping by contextual factors. Together, the two pathways constitute an “individual–context” interactive mechanism of capability generation. The quantitative analysis presented later in this section provides supportive validation for the qualitative findings; its results should be interpreted as evidence of statistical association rather than as tests of causation.

#### The endogenous pathway of individual agency.

The endogenous pathway describes how sales personnel actively construct triadic capabilities through proactive learning, cognitive transfer, and iterative development. The qualitative data point to four interrelated facets of this pathway: starting-point effects of individual background, the role of active learning, cognitive transfer across domains, and temporal patterns of iterative development.

Starting-point effects of individual background. Professional background and prior career experience appear to shape the initial direction of capability development. Interviewees with a marketing background tended to begin with sales capability and gradually extend toward data analytics. As Interviewee #16 recalled, “Coming from a marketing background, I initially focused on customer development, and only later picked up data-analytic skills.” Those with an electrical-engineering background, by contrast, tended to enter the field through digital capability before building communication skills, as Interviewee #22 noted: “With my background in power systems, I started by learning data modeling for electricity, and only later worked on sales communication.” This pattern extends the argument that professional background influences capability development [58], by clarifying its starting-point orientation role in a multidimensional capability setting.

The role of active learning. Sales personnel expand their capabilities across dimensions through multiple forms of active learning, including observational learning (modeling high-performing colleagues), reflective practice (analyzing failure cases), and systematic training (participating in digitalization courses) [55]. Interviewee #7 described this transition vividly: “Seeing colleagues win clients through data analysis, I voluntarily enrolled in the company’s BI training, and within six months I could conduct electricity-load forecasting on my own.” Such accounts indicate that active learning serves as a key mechanism for cross-dimensional capability development.

Cognitive transfer across capability domains. The cognitive frame established by an individual’s initial capability appears to facilitate the acquisition of subsequent capabilities. Sales personnel with strong service capability, for instance, were better attuned to “the customer pain point behind the data.” As Interviewee #13 put it, “People with a service mindset know that customers care about electricity costs, so when they analyze data they focus on peak-valley price differentials.” This form of cognitive transfer extends accounts of absorptive capacity [59] by pointing to a micro-cognitive mechanism through which individuals integrate capabilities across domains.

Temporal patterns of iterative development. Coding of capability-development timelines reveals a four-stage temporal pattern of capability generation, reflecting an evolution from a single-capability focus to multi-capability synergy. Across the interviews, this temporal pattern broadly tracks individuals’ career-development cycles: a focus on initial capability activation within the first year of employment, cross-capability enhancement in the second and third years, synergistic integration in years three to five, and system-level adaptation in later stages of professional development. The detailed four-stage characterization is provided in Table 9.

**Table 9:** Temporal pattern of triadic capability generation and evolution.

Temporal Stage	Core Capability Characteristics	Typical Behavioral Manifestations	Representative First-Order Concepts
**Initial activation**	Single-capability focus; development of foundational competence	Acquisition of product knowledge; introductory sales training	Early-stage single-capability focus; establishing foundation
**Cross-enhancement**	Emergent secondary capability; reflection on capability gaps	Service-driven customer retention; basic data analytics	Awareness of secondary needs; reflection on deficiencies
**Synergistic integration**	Multi-capability convergence; synergistic functioning	Data-informed solution optimization; service-enabled data acquisition	Multi-capability practice; combined deployment of capabilities
**System adaptation**	Dynamic capability reconfiguration; contextual adaptation	Customer-specific allocation of sales, service, and data resources	Contextual adaptability; strategic flexibility

#### The external pathway of contextual shaping.

Alongside the endogenous pathway, the development of triadic capabilities is also shaped by external contextual conditions, reflecting a process of contextual adaptation [60]. Drawing on the qualitative material and supported by the survey data, this section examines how four contextual factors—market digitalization, organizational strategic positioning, competitive-differentiation foundation, and customer-relationship complexity—relate to the differentiation among the three developmental pathways.

Qualitative associations between contexts and pathways. Mapping first-order concepts onto contextual variables revealed systematic associations between contextual configurations and pathway selection. Where the market environment was highly digitalized and the organizational strategy was technology-oriented, the analytics-driven pathway was the dominant trajectory, as observed in CGN New Energy Shenzhen. Where customer relationships were complex and the strategy was service-oriented, the relationship-driven pathway prevailed, as observed in the key-account team of CGN New Energy Guangxi. Where digitalization remained limited and the strategy emphasized market expansion, the market-driven pathway predominated, as observed in the regional development team of CGN New Energy Guangxi. The full qualitative association matrix is provided in S2 Table.

Supportive evidence from the survey data. Multiple regression analysis on the 146 valid questionnaire responses lends further support to the qualitatively identified context–pathway associations. Market digitalization and technology-oriented strategy showed the strongest associations with the analytics-driven pathway; customer-relationship complexity and service-oriented strategy showed the strongest associations with the relationship-driven pathway; and market-expansion strategy showed a positive association with the market-driven pathway, while market digitalization showed a negative association. The full regression results are provided in Table 10. These results are interpreted as supportive evidence of statistical association for the qualitatively identified patterns, rather than as tests of causation.

**Table 10:** Regression analysis results for influencing factors on capability pathways.

Predictor Variables	Analytics Pathway	Relationship Pathway	Market Pathway
Market Digitalization Level	0.38^***^	0.12	−0.23^*^
Org. Orientation (Technology)	0.31^**^	−0.08	−0.11
Org. Orientation (Service)	−0.09	0.34^***^	−0.07
Org. Orientation (Market)	−0.13	−0.15	0.36^***^
Competitive Differentiation Base	0.26^*^	0.29^**^	0.25^*^
Customer Relationship Complexity	−0.14	0.42^***^	−0.19^*^
R^2^	0.32	0.38	0.30
F	16.58^***^	21.63^***^	15.12^***^

*Note:* ^*^ *p* < 0.05, ^**^ *p* < 0.01, ^***^ *p* < 0.001. All coefficients are standardized Beta values (*N* = 146).

Atypical cases and the necessary-but-not-sufficient role of context. To examine the robustness of the context–pathway associations, we analyzed 12 atypical cases—for instance, instances where a relationship-driven pathway occurred in a highly digitalized context. In such cases, insufficient organizational support (e.g., limited access to data-analytic tools) and individual background constraints (e.g., lack of prior data-analytic experience) appeared as the main interfering factors. This pattern suggests that contextual factors function as necessary but not sufficient conditions for pathway selection: their effect is conditioned by individual-level factors. The detailed analysis of these atypical cases is provided in S3 Table.

Combined effects of contextual factor configurations. Further analysis indicates that combinations of contextual factors relate to pathway selection in ways that exceed the contribution of any single factor. Particular configurations appear to alter both the opportunities for learning available to individuals and the perceived value of different capability dimensions, thereby shaping the developmental trajectories that individuals are most likely to follow. The detailed cross-influence analysis is provided in S4 Table.

## Conclusions and discussion

### Principal research findings

Situated within the context of China’s electricity market reform, this study draws on grounded theory and a dual-case design (CGN New Energy Guangxi and CGN New Energy Shenzhen) to investigate the generation and evolution of sales personnel’s triadic capabilities among individuals transitioning from production to sales roles in power-generation enterprises. The principal findings can be summarized along four interrelated dimensions.

#### From a dual to a triadic capability structure.

Under conditions of digital transformation, sales personnel capability is better understood as a triadic structure of “sales–service–digital” capabilities, rather than the conventional “sales–service” duality [7,18]. Digital capability is not an auxiliary instrument supporting sales or service activities [9]; rather, it constitutes an independent core dimension organized around a “data acquisition–analysis–decision transformation” logic. Together with service capability (oriented toward customer problem-solving and experience creation) and sales capability (oriented toward value delivery and transaction closure), digital capability forms a triadic configuration that better reflects the demands of data-driven sales work in the post-reform electricity market.

#### A foundation-innovation dual-layer structure.

Each of the three capabilities exhibits a foundation-layer / innovation-layer structure. The foundation layer captures standardized execution skills, such as basic data collection within digital capability, standardized fault response within service capability, and basic product presentation within sales capability. The innovation layer captures contextualized and forward-looking practices, such as load forecasting within digital capability, customized green-electricity solution design within service capability, and integrated energy-solution selling within sales capability. The two layers are not parallel options but progressively related: foundation-layer proficiency serves as a prerequisite for innovation-layer development, suggesting that capability evolution involves a transition from quantitative accumulation to qualitative transformation. This finding addresses a relatively underspecified aspect of the “operational routines / dynamic capabilities” framework [55] concerning the inter-level connection mechanism.

#### Three differentiated developmental pathways.

Moving beyond the singular linear sequence often assumed in prior research [8,20], this study identifies three differentiated developmental pathways, each associated with distinct contextual conditions.

The analytics-driven pathway (digital → sales → service) is more often observed in highly digitalized, technology-oriented contexts, where capability development begins with data analytics and gradually extends into refined selling and personalized service. The relationship-driven pathway (service → digital → sales) is more often observed in contexts characterized by less digitalized markets and high customer-relationship complexity, where trust built through service provides access to customer data, which in turn supports more targeted selling. The market-driven pathway (sales → service → digital) is more often observed in market-expansion contexts with a broad customer base, where capability development begins with customer acquisition and gradually moves through service-based retention toward data-informed resource optimization.

The three pathways nonetheless converge toward an integrated triadic capability configuration, suggesting a pattern of multi-path convergence: different starting points and developmental sequences may ultimately yield similar capability outcomes. Pathway selection is itself shaped by both initial capabilities and contextual conditions, which is broadly consistent with views on the environmental adaptability of capability development [33].

#### A dual-pathway mechanism of capability generation.

The generation of triadic capabilities is best understood through a dual-pathway mechanism of “endogenous development and contextual shaping.” The endogenous pathway centers on individual agency, manifesting in the starting-point effects of professional background, the role of active learning in cross-dimensional expansion, and the role of cognitive transfer in capability integration. Across the interviews, these elements unfolded along a four-stage temporal pattern moving from initial capability activation through cross-capability enhancement and synergistic integration toward system-level adaptation. The external pathway centers on contextual shaping, with four salient factors identified in the data: market digitalization, organizational strategic positioning, competitive-differentiation foundation, and customer-relationship complexity. The regression results provide supportive evidence for the associations between these contextual factors and pathway selection, broadly consistent with the applicability of the contingency perspective to sales-capability research [26].

### Theoretical contributions and managerial implications

#### Theoretical contributions.

Building on prior work in sales-capability theory and dynamic capability theory, this study advances the literature along three dimensions.

First, a structural extension of sales-service ambidexterity theory. Existing sales-service ambidexterity frameworks [7,18] tend to treat digital elements as auxiliary tools [9]. Through construct independence tests, this study positions digital capability as an independent core dimension alongside sales and service capabilities, and accordingly proposes a “sales–service–digital” triadic capability structure. This finding speaks to the reshaping of the sales role under digital transformation and offers a more contemporary analytical framework for subsequent research.

Second, a micro-level extension of dynamic capability theory. Dynamic capability theory has long centered on the organizational level [55,56]. By shifting the unit of analysis to the individual salesperson, this study identifies a “foundation-layer / innovation-layer” dual structure, specifying a micro-mechanism through which “operational routines” connect with “dynamic capabilities”: foundation-layer proficiency serves as a prerequisite for innovation-layer development, suggesting that capability evolution follows an internal logic of “quantitative accumulation toward qualitative leap.”

Third, a qualification of the linearity assumption in capability development. Prior research has often adopted a single linear developmental path [8]. Across the analytics-driven, relationship-driven, and market-driven contexts examined in this study, capability development trajectories were differentiated, supporting a new proposition of “multi-path convergence”: different initial pathways may, through the combined operation of contextual shaping and cognitive transfer, ultimately yield similar capability syntheses. This finding suggests that capability development is unlikely to follow a universally optimal pathway and takes diverse forms aligned with different contexts, enriching the body of contextualized capability research.

#### Managerial implications.

The findings point to three implications for the management of sales personnel capability development in transitioning enterprises.

Differentiated capability development strategies. Organizations may benefit from aligning capability development with the contextual characteristics they face. In highly digitalized markets, training resources could prioritize data analytics and BI-tool applications to support the analytics-driven pathway. In markets characterized by complex customer relationships, training could emphasize customer relationship management and service-solution design to support the relationship-driven pathway. In market-expansion phases, training could focus on sales techniques and customer acquisition to support the market-driven pathway, while preserving the flexibility to incorporate digital capability as the market matures.

A tiered training system for the triadic capabilities. Training programs could be structured progressively across the foundation and innovation layers. Foundation-level modules could focus on standardized skills, such as data collection and basic service procedures, particularly for newly transitioning employees. Innovation-level modules could emphasize contextualized applications, such as load-forecasting modelling and customized energy solutions, for experienced staff. Cross-dimensional learning modules, such as joint case studies of data-informed service design, may help promote capability synergy in line with prior suggestions on multi-dimensional capability integration [61].

Collaborative mechanisms across functions. Cross-departmental collaboration platforms, such as joint project teams involving sales, technology, and customer-service functions, can foster data sharing, service complementarity, and sales synergy. Knowledge-management systems may help capture and disseminate successful instances of integrated capability deployment. Capability-oriented evaluation systems, incorporating data-driven decision-making and service innovation as performance indicators, can support sales personnel’s transition from a single-skill orientation toward an integrated capability profile, contributing to enhanced customer value creation [30].

### Research limitations and future directions

#### Research limitations.

This study has certain limitations at the methodological, sampling, and analytical levels.

Methodological. The study relies primarily on cross-sectional and retrospective interview data, without multi-year longitudinal tracking. The temporal identification of capability evolution is therefore constrained, and the conditions and timing of pathway transitions cannot be systematically characterized.

Sampling. The study focuses on two subsidiaries within the renewable-energy sector, yielding a relatively narrow sample scope. The generalizability of the findings to other power sub-sectors, such as thermal power and hydropower, and to regions at different stages of marketization, remains to be examined.

Analytical. The unit of analysis is primarily situated at the individual level, and multi-level factors—such as individual traits, organizational culture, and team climate—have not been systematically incorporated. The layered explanation of capability generation mechanisms therefore warrants further development.

#### Future research direction.

Building on these limitations and the frontier issues they bring into view, future research could be advanced along four directions.

Longitudinal studies. A 1–3 year longitudinal design, combined with objective indicators such as training performance, sales outcomes, and customer evaluations, could help identify the conditions of pathway transitions—for instance, how contextual shifts are associated with movement from a market-driven toward an analytics-driven pathway, and the timing and triggers of the foundation-layer to innovation-layer transition.

Cross-industry comparative studies. Future research could extend to traditional thermal-power and hydropower enterprises, as well as to regions at varying stages of marketization, to examine the industry adaptability of the triadic capability structure and its developmental pathways. Broader extensions to other public-utility sectors undergoing market-oriented transitions, such as gas and water, could examine the cross-industry generalizability of the capability structure and provide a more comparatively grounded basis for contextualized capability theory.

Multi-level interaction studies. Future research could introduce moderating variables such as individual learning capacity and organizational support intensity, and adopt an individual–team–organization multi-level analytical framework to examine cross-level interactions—for instance, how organizational strategy is associated with individual capability formation through team climate—thereby deepening the layered understanding of capability generation.

Capability–performance associations and extensions in the AI context. This study centers on how the triadic capabilities emerge and has not systematically examined their association with sales performance. Future research could examine how the triadic capabilities, their dual-layer structure, and their developmental pathways relate to performance outcomes such as customer retention, sales revenue, and customer value creation, and identify which capability combinations are associated with the most pronounced synergistic effects under varying contexts. At a more frontier-oriented level, the rapid diffusion of generative AI tools is reshaping sales practice and shifting the substance of digital capability from a “data–insight–decision” chain toward human–AI collaborative decision-making. How sales personnel establish cognitive complementarity with AI tools, and how the innovation layer within the dual structure is redefined under AI augmentation, constitute forward-looking research questions that open a promising agenda for the further extension of contextualized capability theory.

## Supporting information

S1 Fig The data distillation process from raw text to the first-order concept. This flowchart illustrates the systematic grounding path from qualitative raw interview data, through initial concepts, to the aggregated dimension of data analytical capability.
(TIF)

S2 Fig The axial coding network of the triadic capability framework. A relational structure graph capturing the logical connections and sub-capacities surrounding sales capabilities, service capabilities, and digital capabilities.
(TIF)

S3 Fig Triadic capability structure and evolutionary model for sales personnel. A comprehensive theoretical framework illustrating the interaction between the dual-layer structure and the three developmental pathways.
(TIF)

S1 Table Axial coding results and complete mapping from first-order concepts to aggregate dimensions. The comprehensive grounded theory codebook entries extracted from the dual-case raw text.
(DOCX)

S2 Table Qualitative association matrix between contextual factor configurations and developmental pathways. A cross-case matrix tracing the boundary conditions of analytics-driven, relationship-driven, and market-driven pathways based on 155 coding instances.
(DOCX)

S3 Table Detailed analysis of interfering factors in atypical cases. A diagnostic table profiling the core interfering factors (such as organizational resource allocation and top management decisions) that deviate from standard contextual alignments across the six atypical case instances.
(DOCX)

S4 Table Cross-influence configuration matrix of contextual drivers. An integrative table mapping the non-linear combined effects of digitalization, organizational strategy, and customer relationship complexity on pathway choices across distinct regional sales teams.
(DOCX)

S1 File Survey questionnaire. The questionnaire used in the supportive quantitative component of the study.
(PDF)

S2 File Semi-structured interview guide. The interview guide used for qualitative data collection.
(PDF)

S3 File Coding evidence appendix. An appendix containing anonymized interview quotations and their theoretical mappings.
(PDF)
