# Supplementary material for: Triadic capabilities of sales personnel in power generation enterprises under China’s electricity market reform: Generation and evolution mechanisms
Source: PLoS One. 2026 Aug 10;21(8):e0355359. doi: 10.1371/journal.pone.0355359 (PMC13456286; doi:10.1371/journal.pone.0355359)
Supplement: Supplementary file 1 [file pone.0355359.s008.pdf]

Survey Questionnaire
The Sales-Service-Digitalization Triadic Capabilities Framework for Electric Power Sales Professionals

Research Information for Participants

Dear Participant,

You are cordially invited to participate in this research survey investigating the capability framework of electric power sales professionals. This questionnaire is designed based on rigorous theoretical foundations to explore the core capability architecture of sales personnel within the context of electricity market liberalization.

Research Protocol: This survey is conducted anonymously with strict confidentiality protocols. All data will be used exclusively for academic research purposes. The estimated completion time is 15-20 minutes. Your participation will provide valuable insights for human capital development research in the electric power industry.

Thank you for your support and cooperation.

Section 1: Demographic Information

Please select the option that best describes your situation:

Gender: ☐ Male ☐ Female

Age: ☐ 25-30 ☐ 31-35 ☐ 36-40 ☐ 41-45 ☐ 46-50 ☐ Above 50

Educational Background: ☐ Associate degree or below ☐ Bachelor's degree ☐ Master's degree ☐ Doctoral degree

Professional Background: ☐ Power Engineering ☐ Marketing Management ☐ Information Technology ☐ Other

Sales Experience: ☐ Less than 3 years ☐ 3-7 years ☐ More than 7 years

Entry Mode: ☐ Transferred from production position ☐ Direct entry into sales position

Primary Market Region: ☐ Tier 1-2 cities ☐ Tier 3-4 cities ☐ County-level markets

---

Section 2: Core Capability Assessment

Instructions: Please rate your actual capability level by checking the appropriate number (1=Strongly Disagree, 2=Disagree, 3=Neutral, 4=Agree, 5=Strongly Agree)

A. Sales Capability

A1. I can accurately identify customers' core electricity needs and latent pain points
☐1 ☐2 ☐3 ☐4 ☐5

A2. I can clearly articulate the unique value proposition of our electricity products versus

competitors
☐1 ☐2 ☐3 ☐4 ☐5

A3. I possess effective negotiation capabilities in complex electricity transactions
☐1 ☐2 ☐3 ☐4 ☐5

A4. I can appropriately handle customer objections and advance contract closure
☐1 ☐2 ☐3 ☐4 ☐5

A5. I can coordinate internal resources (technical, financial, etc.) to support sales objectives
☐1 ☐2 ☐3 ☐4 ☐5

A6. I can flexibly adapt sales strategies for customers across different industries
☐1 ☐2 ☐3 ☐4 ☐5

B. Service Capability

B1. I consistently prioritize customer electricity needs in decision-making processes
☐1 ☐2 ☐3 ☐4 ☐5

B2. I can rapidly diagnose and resolve customers' technical electricity issues
☐1 ☐2 ☐3 ☐4 ☐5

B3. I can design customized comprehensive electricity solutions for clients
☐1 ☐2 ☐3 ☐4 ☐5

B4. I can integrate diverse products (wind, solar, etc.) to provide one-stop services
☐1 ☐2 ☐3 ☐4 ☐5

B5. I can continuously optimize service processes and content based on customer feedback
☐1 ☐2 ☐3 ☐4 ☐5

B6. I focus on establishing long-term partnership relationships beyond transactional interactions
☐1 ☐2 ☐3 ☐4 ☐5

C. Digitalization Capability

C1. I can systematically collect multidimensional customer data (load profiles, costs, etc.)
☐1 ☐2 ☐3 ☐4 ☐5

C2. I can analyze customer electricity consumption curves to identify demand patterns and anomalies
☐1 ☐2 ☐3 ☐4 ☐5

C3. I can utilize BI tools and similar technologies for advanced data mining and modeling
☐1 ☐2 ☐3 ☐4 ☐5

C4. I can formulate precise marketing strategies based on data analysis results
☐1 ☐2 ☐3 ☐4 ☐5

C5. I can develop electricity demand forecasting models to support prospective decision-making

☐1 ☐2 ☐3 ☐4 ☐5

C6. I can transform data insights into visualized reports for client presentations
☐1 ☐2 ☐3 ☐4 ☐5

Section 3: Capability Hierarchy Assessment

D. Foundational Capability

D1. I have mastered fundamental promotion and standardized introduction of electricity products
☐1 ☐2 ☐3 ☐4 ☐5

D2. I can respond to and process routine customer service requests following standard procedures
☐1 ☐2 ☐3 ☐4 ☐5

D3. I can conduct basic data collection, organization, and descriptive statistical analysis
☐1 ☐2 ☐3 ☐4 ☐5

E. Innovation Capability

E1. I can innovatively design integrated energy solutions and coordinate cross-departmental resources
☐1 ☐2 ☐3 ☐4 ☐5

E2. I can proactively anticipate customer need evolution and provide forward-looking service proposals
☐1 ☐2 ☐3 ☐4 ☐5

E3. I can employ advanced techniques (predictive modeling, etc.) to guide strategic business decisions
☐1 ☐2 ☐3 ☐4 ☐5

Section 4: Capability Development Pathway

F. Development Pathway Selection

F1. My triad capability development primarily follows which temporal pathway?

☐ Analytics-Driven Pathway: Digitalization → Sales → Service
☐ Relationship-Driven Pathway: Service → Digitalization → Sales
☐ Market-Driven Pathway: Sales → Service → Digitalization
☐ Synergistic Development Pathway: Simultaneous evolution of all three competencies

G. Capability Synergy Relationships

G1. Enhancement of sales capability significantly promotes my service capability development

☐1 ☐2 ☐3 ☐4 ☐5

G2. Enhancement of service capability significantly promotes my digitalization capability development
☐1 ☐2 ☐3 ☐4 ☐5

G3. Enhancement of digitalization capability significantly promotes my sales capability development
☐1 ☐2 ☐3 ☐4 ☐5

G4. All three competencies must be synergistically applied in complex customer scenarios for success
☐1 ☐2 ☐3 ☐4 ☐5

G5. I can effectively integrate the triad competencies to form distinctive competitive advantages
☐1 ☐2 ☐3 ☐4 ☐5

Section 5: Development Stage Assessment

H1. I experienced a distinct initial capability establishment phase (focused on mastering a single core capability)
☐1 ☐2 ☐3 ☐4 ☐5

H2. I experienced a capability cross-enhancement phase (recognized the need to develop a second capability)
☐1 ☐2 ☐3 ☐4 ☐5

H3. I experienced a capability synergistic integration phase (multiple competencies began to merge in application)
☐1 ☐2 ☐3 ☐4 ☐5

H4. I am currently in a capability system optimization phase (flexibly adjusting capability combinations to adapt to different contexts)
☐1 ☐2 ☐3 ☐4 ☐5

Section 6: Capability Integration Mechanisms

I. Capability Complementarity Mechanism

I1. Application of digitalization capability significantly enhances my sales precision and persuasiveness
☐1 ☐2 ☐3 ☐4 ☐5

I2. High-quality service performance helps me acquire richer and more accurate customer data
☐1 ☐2 ☐3 ☐4 ☐5

I3. Successful sales activities provide me with more opportunities for in-depth customer service
☐1 ☐2 ☐3 ☐4 ☐5

J. Cognitive Transfer Mechanism

J1. I can effectively apply successful experiences from one capability to learning other competencies
☐1 ☐2 ☐3 ☐4 ☐5

J2. Thinking patterns formed in one capability domain facilitate rapid mastery of other competencies
☐1 ☐2 ☐3 ☐4 ☐5

J3. I can identify similarities among different competencies to accelerate the learning process
☐1 ☐2 ☐3 ☐4 ☐5

Section 7: Work Environment Influencing Factors

K. Market Environment

K1. The digitalization level of my market requires sales personnel to possess data analytics capabilities
☐1 ☐2 ☐3 ☐4 ☐5

K2. Intense competition in my market compels me to develop differentiated comprehensive competencies
☐1 ☐2 ☐3 ☐4 ☐5

L. Organizational Environment

L1. The company's technology-oriented strategy drives me to prioritize digitalization capability development
☐1 ☐2 ☐3 ☐4 ☐5

L2. The company's training system and digital tools effectively support my capability development
☐1 ☐2 ☐3 ☐4 ☐5

L3. The company's culture encouraging cross-departmental collaboration promotes my capability integration
☐1 ☐2 ☐3 ☐4 ☐5

M. Customer Environment

M1. The complexity of my customer relationships requires high-level service capability
☐1 ☐2 ☐3 ☐4 ☐5

M2. My customers have high expectations for data-supported decision recommendations
☐1 ☐2 ☐3 ☐4 ☐5

M3. The personalization level of my customer needs drives continuous innovation in service solutions
☐1 ☐2 ☐3 ☐4 ☐5

Section 8: Development Pathway Characteristics

N. Path Dependency

N1. The initially developed capability type significantly influences subsequent capability learning efficiency
☐1 ☐2 ☐3 ☐4 ☐5

N2. My capability development pathway is strongly influenced by professional background and work experience
☐1 ☐2 ☐3 ☐4 ☐5

N3. Changing an established capability development pathway requires additional learning costs
☐1 ☐2 ☐3 ☐4 ☐5

O. Path Convergence

O1. Regardless of which capability initiates development, synergistic application of all three is ultimately required
☐1 ☐2 ☐3 ☐4 ☐5

O2. Colleagues with different development pathways eventually converge to similar comprehensive capability levels
☐1 ☐2 ☐3 ☐4 ☐5

O3. Long-term market requirements necessitate that all sales personnel develop toward triad capability
☐1 ☐2 ☐3 ☐4 ☐5

Section 9: Work Performance Assessment

P1. My sales performance ranks among the leading levels within my peer group
☐1 ☐2 ☐3 ☐4 ☐5

P2. The quality and trust level of relationships I establish with customers continuously improves
☐1 ☐2 ☐3 ☐4 ☐5

P3. My work efficiency and problem-solving capabilities significantly exceed past performance
☐1 ☐2 ☐3 ☐4 ☐5

P4. My overall work performance receives high recognition from supervisors and customers

☐1 ☐2 ☐3 ☐4 ☐5

End of Questionnaire. Thank you for your participation!
