# Supplementary material for: Triadic capabilities of sales personnel in power generation enterprises under China’s electricity market reform: Generation and evolution mechanisms
Source: PLoS One. 2026 Aug 10;21(8):e0355359. doi: 10.1371/journal.pone.0355359 (PMC13456286; doi:10.1371/journal.pone.0355359)
Supplement: Supplementary file 2 [file pone.0355359.s009.pdf]

# Semi-structured Interview Guide: Sales Personnel Capability Evolution in China's Electricity Market Reform Context

## 1.Theoretical Foundation and Research Framework

### 1.1 Research Objectives

This study explores the mechanisms, pathways, and influencing factors of sales personnel capability evolution within China's electricity market reform context.

### 1.2 Theoretical Foundations

Capability Evolution Theory: Dynamic development of individual capabilities in response to environmental changes

Organizational Learning Theory: Knowledge acquisition and capability enhancement through organizational processes

Contextual Adaptation Theory: Individual and organizational adaptation to environmental contingencies

### 1.3 Operational Definitions of Core Constructs

Sales Capability: Comprehensive skills required to identify, develop, and maintain customer relationships to achieve sales objectives within the electricity market context.

Service Capability: The ability to understand customer needs and provide customized solutions, particularly relevant to electricity market products and services.

Digital Capability: Proficiency in utilizing digital technologies and data analytics to support decision-making processes in electricity sales and customer management.

Capability Evolution: The dynamic development and adaptation process of individual capabilities within specific contextual environments over time.

## 2. Interview Implementation Protocol

Basic Interview Information

Interview Code: _____________

Date: _____________

Location: _________________

Recording Permission: ☐ Granted ☐ Not Granted

Estimated Duration: 40-60 minutes

Respondent Profile

Anonymized Code: _____________

Demographic Characteristics:

Age Range: ☐ 25-30 ☐ 31-35 ☐ 36-40 ☐ 41-45 ☐ 46-50 ☐ 50+

Gender: ☐ Male ☐ Female ☐ Other

Education Level: ☐ Associate ☐ Bachelor's ☐ Master's ☐ PhD ☐ Other: _______

Professional Background:

Current Position: _________________

Industry Experience: _______ years

Sales Position Experience: _______ years

Role Transition Date: _______ (if applicable)

## 3. Interview Question Framework

*Note: This protocol serves as a semi-structured interview guide. It is designed to ensure comprehensive coverage of central thematic domains rather than to be followed as a rigid script. Interviewers should utilize this framework as a flexible roadmap for dialogue. The primary objective is to guide the conversation organically based on the participant's responses, prioritizing in-depth exploration and probing. The essence of a successful interview lies in active listening and thoughtful follow-up, not in mechanically completing a questionnaire. Practitioners are encouraged to focus on eliciting rich, illustrative narratives and critical insights from participants, adapting the sequence and depth of questioning dynamically as the discourse unfolds.*

### Section A: Role Transition and Cognitive Reconstruction

Research Focus: How do sales personnel perceive and adapt to role transitions within the electricity market reform context?

Primary Questions:

"Please describe the main changes in your work content following your transition from [previous position] to your current sales role."

"How do you understand the impact of electricity market reform on your professional role?"
Probing Questions:
Can you provide specific examples of how these changes manifest in your daily work?
What do you consider the core challenges of this role transition?
How have you adjusted your cognitive frameworks and behaviors to adapt to this new role?
What specific knowledge or skills from your previous role have proven transferable or obsolete?

**Section B: Capability Dimension Evolution Analysis**
B.1 Sales Capability Development
Core Question: "How have your customer development and relationship maintenance capabilities evolved since joining the electricity sales sector?"
Specific Areas of Inquiry:
Describe your most challenging customer development experience and identify the key success factors
How do electricity sales differ from traditional industry sales in terms of required capabilities?
What methods do you use to assess and enhance your sales skills?
How has the regulatory environment influenced your approach to customer acquisition?
B.2 Service Capability Evolution
Core Question: "How has your customer service and solution design capability evolved within the electricity market context?"
Specific Areas of Inquiry:
Share a successful case where you designed a customized solution for a client
How do you balance standardized service offerings with individualized customer needs?
What factors have most significantly contributed to your service capability enhancement?
How do you handle the technical complexity of electricity products when serving customers?
B.3 Digital Capability Development
Core Question: "How have your data analytics and digital tool application capabilities developed since entering the electricity sales sector?"
Specific Areas of Inquiry:
Describe the role of data analysis in your decision-making processes
How have you acquired and mastered relevant digital skills?
What impact has digital capability development had on your work efficiency?
Which digital tools or platforms have been most instrumental in your role?

**Section C: Capability Integration and Synergy Mechanisms**
Research Focus: How do different capability dimensions interact and develop synergistically?
Core Questions:
"Describe a complex work scenario that required simultaneous application of sales, service, and digital capabilities."
"What relationships exist among these three capability dimensions?"
"Which capability serves as the foundation for developing others, and why?"
Deep Exploration:
Do conflicts arise between different capabilities? How do you manage them?
Is there a specific temporal sequence for developing different capabilities?
How do you achieve effective capability integration?
Can you identify critical incidents that accelerated capability integration?

**Section D: Contextual Factors Analysis**
D.1 Market Environment Impact
How does your market's competitive landscape influence your capability requirements?
How do different customer segments vary in their capability demands?
How do policy environment changes affect your capability development direction?
What market trends do you anticipate will reshape capability requirements?

D.2 Organizational Support System
What capability development support does your organization provide, and how effective is it?
How do organizational culture and management systems influence your capability evolution?
What additional organizational support would facilitate your capability development?
How does your organization's strategic orientation affect individual capability priorities?
D.3 Individual Characteristics Impact
How do your educational background and work experience influence your capability development pathway?
How do your learning preferences affect your capability acquisition methods?
What are your primary motivations for capability development?
What personal barriers have you encountered in capability development?

**Section E: Evolution Pathway and Future Prospects**
Temporal Dimension Analysis
"Please review your capability development timeline and describe key turning points or breakthrough moments."
"What new capabilities do you believe sales personnel will need to develop over the next 3-5 years?"
"Based on your experience, what advice would you give to newly recruited sales personnel?"
Trend Forecasting
Which traditional capabilities will maintain their importance, and which might become obsolete?
How do you perceive artificial intelligence's impact on sales work in the electricity sector?
What are your professional development plans and career aspirations?
How might electricity market evolution reshape the sales profession?

**4. Data Quality Control Framework**
Interview Quality Assessment Criteria
Information Richness: ☐ High ☐ Moderate ☐ Low
Response Depth: ☐ Deep ☐ Adequate ☐ Surface-level
Logical Consistency: ☐ Strong ☐ Moderate ☐ Weak
Case Specificity: ☐ Detailed ☐ General ☐ Abstract
Post-Interview Actions
Concepts Requiring Clarification: ________________
Information Requiring Supplementation: ________________
Recommended Follow-up Respondents: ________________
Researcher Reflection Log
Key Insights from Interview Process: ________________
Theoretical Framework Applicability Assessment: ________________
Interview Guide Improvement Recommendations: ________________

**5. Ethical Statement and Informed Consent**
Research Purpose
This study aims to explore the patterns of sales personnel capability evolution within China's electricity market reform context. Research findings will be used for academic publication and policy recommendations.
Privacy Protection
Your personal information will be strictly confidential, with anonymized coding used in research reports.
Voluntary Participation
You have the right to withdraw from the interview at any time or decline to answer specific questions.
Consent Confirmation
☐ agree to participate in this research interview
☐ agree to have the interview recorded (for research purposes only)
☐ agree to have research results used for academic publication (with anonymization)
