# Supplementary material for: Triadic capabilities of sales personnel in power generation enterprises under China’s electricity market reform: Generation and evolution mechanisms
Source: PLoS One. 2026 Aug 10;21(8):e0355359. doi: 10.1371/journal.pone.0355359 (PMC13456286; doi:10.1371/journal.pone.0355359)
Supplement: Supplementary file 3 [file pone.0355359.s010.pdf]

Coding Evidence Appendix: Anonymized Interview Quotes and Theoretical Mapping

Note on Anonymization: In strict accordance with the ethical approval (Approval No. 2025011301) and PLOS ONE's Human Subjects Research policy, all corporate names, specific geographic references, exact temporal markers, proprietary software names, and identifiable personal names have been replaced with generic equivalents. Speaker labels indicate functional affiliation only; no individual is identifiable. The original unredacted transcript is securely stored by the research team subject to corporate non-disclosure agreements.

Theme 1: Role Transition from Production to Sales Positions Quote 1 (Front-line Salesperson, Marketing Center) "I joined the Company in 2012 and have been in marketing for two years, not 21 years. ... I was at our headquarters, moving across several power plants in the southern region." *Corresponding construct:* Empirical evidence of the production-to-sales transition phenomenon. **Quote 2 (Front-line Salesperson, Trading Center)** "I joined the Company right after graduation. Initially in production, then transitioned to power marketing last year." *Corresponding construct:* Generational variation in transition pathways (veteran employees vs. younger technical staff).

Theme 2: Sales Capability (Aggregate Dimension A3) Quote 3 (Front-line Salesperson, Marketing Center) "The biggest difference is interacting with people. In production, we dealt with machines. In marketing, it's more about dealing with people, which requires higher skills." *Corresponding constructs:* Customer Insight Capability; Transaction Completion Capability. **Quote 4 (Front-line Salesperson, Marketing Center)** "There was this perception of informal social engagements to close deals. ... My understanding has changed. While building rapport is important, the core business activities are crucial too." *Corresponding construct:* Value Marketing Capability — cognitive reconstruction from transaction-oriented to value-oriented selling.

Theme 3: Service Capability (Aggregate Dimension A2) Quote 5 (Mid-level Manager, Marketing Center) "Our client base primarily consists of enterprises, so we focus on understanding their power needs and providing tailored services. This involves regular visits and engagement activities to build strong relationships." *Corresponding constructs:* Customer-Centric Service Orientation; Relationship Maintenance Capability. **Quote 6 (Senior Manager, Trading Center) — Critical Incident** "For example, during the peak pandemic period, when everyone was sick, we still maintained customer connections, signing contracts of substantial value despite high fevers. ... Even after your shift, if something comes up, you have to handle it." *Corresponding constructs:* Problem-Solving Capability; Relationship Maintenance Capability. Critical incident illustrating service capability as the core element of the relationship-driven pathway.

Theme 4: Digital Capability (Aggregate Dimension A1) — *Core Concept* Quote 7 (Front-line Salespersons, Trading Center & Marketing Center) "Especially in our business, like using instant messaging platforms ... We use them a lot. They improve efficiency and communication. It's not possible to visit every client daily, so these tools help filter clients and manage communication more thoughtfully." *Corresponding constructs:* Data Acquisition Capability — representing the foundational layer of digital capability (overcoming physical boundaries). **Quote 8 (Front-line Salesperson, Trading Center)** "In the trading center, technical skills remain crucial alongside sales skills and customer interactions. ... Yes, it involves developing pricing and trading strategies, which requires a lot of technical analysis." *Corresponding constructs:* Data Analytics Capability; Decision Application Capability — representing the innovation layer. *Note:* This demonstrates that digital capability is an independent, high-level analytical competence for complex energy trading (Human-AI collaboration), not merely an auxiliary CRM tool.

Theme 5: Differentiated Development Pathways Quote 9 (Mid-level Manager, Marketing Center) "Our department is divided into two sections: market development and daily operations. ... Our marketing center focuses on market development and client acquisition, while the transaction center handles the subsequent power transactions and contract execution." *Corresponding construct:* Pathway differentiation — structural foundation for the relationship-driven vs. analytics-driven pathway distinction. **Quote 10 (Mid-level Manager, Marketing Center & Senior Manager, Trading Center)** "Our marketing team needs to develop market opportunities and manage client relations effectively. ... This is a transitional phase. Ideally, once we have sufficient personnel, clear delineations between roles will be established." *Corresponding construct:* Synergistic capability development stage — evidence of the transitional phase.

Theme 6: Contextual Factors Shaping Capability Pathways Quote 11 (Senior Manager, Trading Center) "Marketing is crucial, as it drives our business growth and client relations. Although our business segment is still expanding, marketing will eventually become the leading force as we stabilize our production capacity." *Corresponding construct:* Organizational strategic positioning — qualitative corroboration linking market-oriented strategy to the market-driven pathway.

Theme 7: Key Critical Incident — Endogenous Development Mechanism Quote 12 (Front-line Salesperson, Marketing Center) "When I was working at the plant, my understanding and perceptions of the outside world, as well as my willingness to learn, were quite limited. I thought that as long as I mastered the technical aspects, I would surely gain recognition from the company, leaders, and colleagues, and I would progress. However, after transitioning to a marketing position, especially during a client reception with a senior manager, I realized a significant issue. ... I observed the client's extensive knowledge and deep insights. ... This made me realize that I need to enhance my understanding, broaden my horizons, and improve my information sensitivity." *Corresponding construct:* Endogenous development mechanism /

Cognitive transfer mechanism. A compelling critical incident demonstrating individual cognitive awakening.
