# Supplementary material for: Triadic capabilities of sales personnel in power generation enterprises under China’s electricity market reform: Generation and evolution mechanisms
Source: PLoS One. 2026 Aug 10;21(8):e0355359. doi: 10.1371/journal.pone.0355359 (PMC13456286; doi:10.1371/journal.pone.0355359)
Supplement: Supplementary file 4 [file pone.0355359.s004.docx]

S1 Table :Axial Coding Results (Selected Categories)

| Primary First-Order Concepts (Selected Examples) | Second-Order Themes | Aggregate Dimensions |
| --- | --- | --- |
| Multi-source data integration, data collection strategies, data quality control | N1. Data acquisition capabilities | A1. Digital capabilities |
| Data interpretation requirements, data mining skills, pattern recognition abilities | N2. Data analytics capabilities |  |
| Graphical presentation, data storytelling, visualization tool deployment | N3. Data visualization capabilities |  |
| Data-driven decision making, predictive decision processes, evidence-based solution validation | N4. Decision application capabilities |  |
| Service-oriented transformation, problem-solving prioritization, customer experience focus | N5. Customer-centric service orientation | A2. Service capabilities |
| Problem diagnosis skills, rapid response mechanisms, demand sensitivity | N6. Problem-solving capabilities |  |
| Trust-building behaviors, long-term relationship cultivation, customer communication competencies | N7. Relationship maintenance capabilities |  |
| Personalized solution design, customized service delivery, differentiated service manifestation | N8. Solution design capabilities |  |
| Precise customer targeting, value proposition identification, opportunity screening capabilities | N9. Customer insight capabilities | A3. Sales capabilities |
| Value-based selling techniques, value proposition articulation, differentiation positioning | N10. Value marketing capabilities |  |
| Negotiation strategy implementation, objection handling techniques, deal closure capabilities | N11. Transaction completion capabilities |  |
| Cross-functional coordination, resource mobilization abilities, collaborative selling | N12. Sales resource integration |  |
| Data-enhanced selling, service-enriched data generation, sales-facilitated service delivery | N13. Capability complementarity mechanisms | A4. Capability evolution dynamics |
| Knowledge integration processes, practice convergence pathways, cognitive framework transformation | N14. Capability integration mechanisms |  |
| Initial single-capability focus, onboarding skill training emphasis, core competency establishment phase | N15. Initial capability activation | A5. Capability development stages |
| Secondary capability awareness emergence, competency gap reflection, capability complementarity experimentation | N16. Capability cross-enhancement |  |
| Multi-capability integration practice, capability interaction perception, comprehensive competency utilization | N17. Capability synergistic integration |  |
| Contextual adaptation abilities, flexible strategy adjustment, multi-dimensional capability configuration | N18. Capability system optimization |  |
| Data accessibility, customer digital adoption readiness, market transparency levels | N19. Market digitalization degree | A6. Contextual factors |
| Market share strategy, customer value strategy, technological leadership strategy | N20. Organizational strategic positioning |  |
| Price differentiation, service differentiation, solution differentiation | N21. Competitive differentiation foundations |  |
| Transactional relationships, consultative relationships, collaborative relationships, relationship hierarchy diversity | N22. Customer relationship complexity |  |
| Differentiated training design, experiential learning opportunities, mentorship system establishment | N23. Capability development mechanisms | A7. Organizational factors |
| Multi-dimensional performance evaluation, phased incentive strategies, capability-oriented assessment | N24. Evaluation and incentive mechanisms |  |
