# Supplementary material for: Triadic capabilities of sales personnel in power generation enterprises under China’s electricity market reform: Generation and evolution mechanisms
Source: PLoS One. 2026 Aug 10;21(8):e0355359. doi: 10.1371/journal.pone.0355359 (PMC13456286; doi:10.1371/journal.pone.0355359)
Supplement: Supplementary file 5 [file pone.0355359.s005.docx]

S2 Table :Qualitative Association Matrix between Contextual Factors and Capability Development Pathways

| contextual Factor Combinations | Analytics-Driven Pathway (Frequency) | Relationship-Driven Pathway (Frequency) | Market-Driven Pathway (Frequency) | Association Strength Test |
| --- | --- | --- | --- | --- |
| High Digitalization + Technology Orientation | 42 | 8 | 6 | χ²=36.5, p<0.01 |
| High Relational Complexity + Service Orientation | 7 | 35 | 5 | χ²=32.7, p<0.01 |
| Low Digitalization + Market Expansion | 5 | 7 | 38 | χ²=34.2, p<0.01 |

*Note: Frequencies are based on interview coding statistics (N=155). Association strength was verified through chi-square tests.*
