# Supplementary material for: Triadic capabilities of sales personnel in power generation enterprises under China’s electricity market reform: Generation and evolution mechanisms
Source: PLoS One. 2026 Aug 10;21(8):e0355359. doi: 10.1371/journal.pone.0355359 (PMC13456286; doi:10.1371/journal.pone.0355359)
Supplement: Supplementary file 6 [file pone.0355359.s006.docx]

S3 Table :Analysis of Interfering Factors in Atypical Cases

| Atypical Case Type | Number of Cases | Core Interfering Factors | Theoretical Implications |
| --- | --- | --- | --- |
| High Digitalization Context → Relationship-Driven Pathway | 3 | Absence of organizational data tools; Strong individual service background | Organizational resource allocation can moderate the impact of context on pathway selection |
| High Relational Complexity Context → Analytics-Driven Pathway | 2 | Customer demands for data support; Mandatory organizational data assessment | Customer requirements and organizational systems can alter pathway choices |
| Low Digitalization Context → Analytics-Driven Pathway | 1 | Leadership emphasis; Strategic transformation pilot program | Top management decisions can temporarily overcome contextual constraints |
