# Supplementary material for: Triadic capabilities of sales personnel in power generation enterprises under China’s electricity market reform: Generation and evolution mechanisms
Source: PLoS One. 2026 Aug 10;21(8):e0355359. doi: 10.1371/journal.pone.0355359 (PMC13456286; doi:10.1371/journal.pone.0355359)
Supplement: Supplementary file 7 [file pone.0355359.s007.docx]

S4 Table : Cross-Influence Effects of Contextual Factor Combinations

| Contextual Factor Combination | Dominant Pathway | Representative Cases | Number of Cases |
| --- | --- | --- | --- |
| High Digitalization + Technology Orientation + Product Differentiation | Analytics-Driven | Technology-focused sales teams (Region A) | 3 |
| High Relational Complexity + Customer Value Orientation + Service Differentiation | Relationship-Driven | Key account management teams (Region B) | 5 |
| Low Digitalization + Market Share Strategy + Transactional Customers | Market-Driven | Territory expansion teams (Region C) | 2 |
